# Activating KIR2DS4 Is Expressed by Uterine NK Cells and Contributes to Successful Pregnancy

**DOI:** 10.4049/jimmunol.1601279

**Published:** 2016-11-04

**Authors:** Philippa R. Kennedy, Olympe Chazara, Lucy Gardner, Martin A. Ivarsson, Lydia E. Farrell, Shiqiu Xiong, Susan E. Hiby, Francesco Colucci, Andrew M. Sharkey, Ashley Moffett

**Affiliations:** * Department of Pathology, University of Cambridge, Cambridge CB2 1QP, United Kingdom;; † Centre for Trophoblast Research, University of Cambridge, Cambridge CB2 3EG, United Kingdom;; ‡ Manchester Collaborative Centre for Inflammation Research, University of Manchester, Manchester M13 9NT, United Kingdom; § Department of Molecular and Cell Biology, University of Leicester, Leicester LE1 7RH, United Kingdom; and; ¶ Department of Obstetrics and Gynaecology, University of Cambridge School of Clinical Medicine, National Institute for Health Research Cambridge Biomedical Research Centre, Addenbrooke’s Hospital, Cambridge CB2 0SP, United Kingdom

## Abstract

Tissue-specific NK cells are abundant in the pregnant uterus and interact with invading placental trophoblast cells that transform the maternal arteries to increase the fetoplacental blood supply. Genetic case-control studies have implicated killer cell Ig-like receptor (KIR) genes and their *HLA* ligands in pregnancy disorders characterized by failure of trophoblast arterial transformation. Activating *KIR2DS1* or *KIR2DS5* (when located in the centromeric region as in Africans) lower the risk of disorders when there is a fetal *HLA-C* allele carrying a C2 epitope. In this study, we investigated another activating KIR, *KIR2DS4*, and provide genetic evidence for a similar effect when carried with *KIR2DS1*. KIR2DS4 is expressed by ∼45% of uterine NK (uNK) cells. Similarly to KIR2DS1, triggering of KIR2DS4 on uNK cells led to secretion of GM-CSF and other chemokines, known to promote placental trophoblast invasion. Additionally, XCL1 and CCL1, identified in a screen of 120 different cytokines, were consistently secreted upon activation of KIR2DS4 on uNK cells. Inhibitory *KIR2DL5A*, carried in linkage disequilibrium with *KIR2DS1*, is expressed by peripheral blood NK cells but not by uNK cells, highlighting the unique phenotype of uNK cells compared with peripheral blood NK cells. That KIR2DS4, KIR2DS1, and some alleles of KIR2DS5 contribute to successful pregnancy suggests that activation of uNK cells by KIR binding to HLA-C is a generic mechanism promoting trophoblast invasion into the decidua.

## Introduction

Natural killer cells use a combination of activating and inhibitory receptors to recognize viruses and cancerous cells (1). That the same receptors are also used to recognize fetal cells by tissue-specific uterine NK (uNK) cells (2) indicates two strong contrasting evolutionary pressures, that is, disease resistance and successful reproduction, with both showing evidence of balancing selection (3, 4). NK cells in the pregnant uterus, decidual NK (dNK) cells, are different phenotypically and functionally from peripheral blood NK (pbNK) cells (5–10). Evidence from genetic and functional studies suggests that dNK cells regulate trophoblast transformation of the uterine spiral arteries necessary for increasing the blood supply to the fetoplacental unit until the end of gestation (11–14).

**FIGURE 1.**
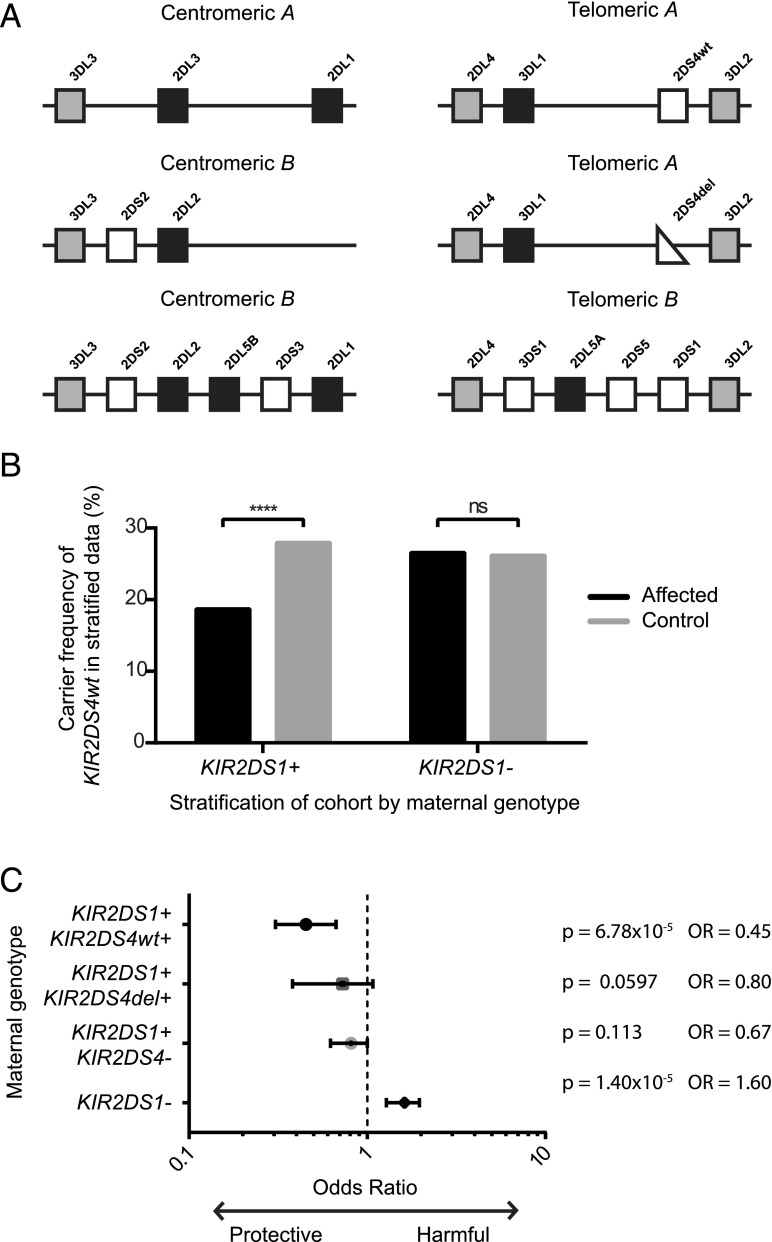
*KIR2DS4wt* in epistasis with *KIR2DS1* is associated with a lower risk of pregnancy disorders. (**A**) The LD blocks that make up >94% of European *KIR* genotypes (17). An individual’s KIR genotype contains two haplotypes, each with one centromeric (left) and one telomeric (right) block. These blocks contain activating (white) and inhibitory (black) genes in LD. Framework genes (gray) are found in all haplotypes. The three most common telomeric blocks contain either *KIR2DS4wt*, *KIR2DS4del*, or *KIR2DS1*. (**B**) Women were stratified according to the presence or absence of the protective gene *KIR2DS1*, as a Breslow–Day test indicated epistasis between *KIR2DS1* and *KIR2DS4wt*. The carrier frequency of *KIR2DS4wt* was then compared between women with affected pregnancies and healthy control pregnancies within each subgroup. The presence of *KIR2DS4wt* was protective (Cochran–Mantel–Haenszel test *p* = 5.7 × 10^−4^, OR = 0.59). (**C**) Then, within the women carrying *KIR2DS1*, the double-positive *KIR2DS1^+^KIR2DS4wt^+^* are the most protected (*p* = 6.78 × 10^−5^, OR = 0.45).

The NK cell receptors particularly implicated in reproductive health are the highly polymorphic killer cell Ig-like receptor (KIR) family (15). A *KIR* genotype is made up of two *KIR* haplotypes that can differ by both gene content and allelic variation. The genes in these haplotypes are so densely clustered on chromosome 19 that they are generally inherited as haplotypic centromeric and telomeric blocks (16, 17) (Fig. 1A). The dominant ligands for KIR are HLA-C allotypes. All individuals have KIRs that will bind to HLA-C allotypes as two groups depending on the C1 or C2 epitope that they bear. There is an increased risk of pregnancy disorders with certain inhibitory maternal *KIR* and fetal *HLA-C* combinations. Case-control genetic studies of Europeans have shown that pregnancy disorders that result from defective placentation with inadequate trophoblast arterial transformation (e.g., pre-eclampsia, fetal growth restriction, and recurrent miscarriage) are linked to an absence of the telomeric *B* (*Tel-B*) *KIR* region in the mother (Fig. 1A) and the presence of paternal *C2* in the fetus (13, 18, 19). In contrast, pregnancies resulting in babies with increased birth weights are also associated with the presence of a paternal *C2* allele in the fetus, but with a maternal *Tel-B KIR* region (20). The tight linkage disequilibrium (LD) of KIRs makes it difficult to determine through genetic studies alone which gene is responsible, so functional studies are required to complement this work.

Of the KIRs in the *Tel-B* region, activating *KIR2DS1* is the most likely candidate for enhancing placentation, because it can bind to C2 allotypes. The inhibitory counterpart, *KIR2DL1*, also binds strongly to C2 allotypes, is present in the centromeric *A* and some centromeric *B* (*Cen-B*) regions, and is carried by ∼98% of individuals. Therefore, in the absence of *KIR2DS1* (55–60% of Europeans), the dominant effect of paternal trophoblast C2 allotypes interacting with dNK cells is inhibition. Ligation of KIR2DS1 on dNK cells induces production of cytokines and chemokines, such as GM-CSF, which can induce trophoblast migration (12). Thus, our current model of pregnancy indicates that when C2 allotypes derived from the father are expressed by trophoblast, KIR2DS1 activates dNK cells to secrete cytokines that encourage deeper invasion of the uterus by trophoblast and promote spiral artery remodeling and a better blood supply for the fetus (2). In the absence of KIR2DS1, insufficient activation of dNK cells results in poor trophoblast invasion, placental stress, growth restriction of the fetus, and pre-eclampsia.

In a similar Ugandan case-control study, we found no protective effect for pre-eclampsia of the *Tel-B* region, including *KIR2DS1* (carried by ∼20% of control women). Instead, certain alleles of an activating *KIR*, *KIR2DS5*, present in *Cen-B* were more frequent in controls compared with pre-eclamptic pregnancies (21). *KIR2DS5* is always located in the *Tel-B* region in non-African populations and is carried in tight LD with *KIR2DS1*. It thus could contribute to the protective effect of *Tel-B* in Europeans, but whether it is expressed or binds C2 allotypes is still controversial. In addition to *KIR2DS1* and *KIR2DS5*, *KIR2DL5A* is also present in *Tel-B* and remains an enigmatic KIR in terms of ligands and functions (22).

Other activating KIRs that might recognize ligands on trophoblast and influence pregnancy outcome include *KIR3DS1* and *KIR2DS2–4*. KIR3DS1, in LD with *KIR2DS1*, binds HLA-B allotypes carrying the Bw4 motif (23), but HLA-B molecules are never expressed by trophoblast (24, 25). KIR2DS2 is predicted to bind the C1 motif through homology with KIR2DL2/3; the presence of fetal C1 alone is always neutral in our genetic case control studies. KIR2DS3 is not expressed at the cell surface (26). This leaves *KIR2DS4*, present in the telomeric *A* (*Tel-A*) region, that occurs either as a truncated (*KIR2DS4del*) (alleles *003/004/006 are carried by ∼80% of Europeans) or full-length (*KIR2DS4wt*) form (allele *001 is carried by ∼35% of Europeans). *KIR2DS4del* has a 22-bp deletion that introduces a frameshift mutation that results in a soluble protein with only one intact Ig-like domain (27). Whereas KIR2DS4wt has been reported to bind some HLA-C alleles carrying both the C1 and C2 epitopes, soluble KIR2DS4del does not bind HLA class I molecules (28). We previously found a negative association of *KIR2DS4del* with pregnancy outcome, but no positive effect of *KIR2DS4wt* (13).

In this study, to investigate the role of KIR other than KIR2DS1 in successful pregnancy, we have studied the expression and function of KIR2DS4 and KIR2DL5 on dNK cells. From this we demonstrate that activation of dNK cells is a general mechanism that is beneficial to pregnancy.

## Materials and Methods

### Primary tissue

Tissue and matched peripheral blood samples were obtained from women undergoing elective terminations in the first trimester of pregnancy; blood was also obtained from healthy volunteers. Both sets of patients gave informed consent. Ethical approval for the use of these tissues was obtained from the Cambridge Local Research Ethics Committee (REC 04/Q0108/23). Leukocytes and placental samples were isolated as previously described (29).

### Cell lines

Cell lines transfected with cDNA for single KIR were used to test Ab specificities. KIR2DL1^+^, KIR2DL3^+^, KIR2DS1^+^, KIR2DS2^+^, KIR2DS4^+^ (30), or KIR3DS1^+^ (31) BWZ cells were the gift of Eric Vivier. KIR2DL2^+^, KIR2DS5^+^, KIR3DL1^+^ (31), or KIR3DL3^+^ (32) BA/F3 cells were the gift of Chiwen Chang, as was cDNA for *KIR2DL5* used to transiently transfect HEK293T cells. KIR2DL4^+^ Jurkat cells were the gift of Kerry Campbell. Paul Norman supplied cDNA of *KIR3DL2* for transient transfection into HEK293T cells.

### Flow cytometry

dNK cells were gated on as live, CD9^+^CD56^+^ cells. pbNK cells were gated on as live CD56^+^CD3^−^ cells. The following Abs were used: Live/Dead discriminator (Life Technologies), CD9 (SN4 or M-L13 from eBioscience or BD Biosciences, respectively), CD56 (HCD56 from BioLegend), and CD3 (SK7) from BD Biosciences. Fibroblasts and macrophages were identified using CD10 (HI10a from BioLegend) and CD14 Abs (MφP9 and HCD14 from BD Pharmingen and BioLegend), respectively. The following Abs were used to stain KIRs: UPR1 (KIR2DL5) from BioLegend and Carlos Vilches (33); 179315 (KIR2DS4), 143211 (KIR2DL1), and 181703 (KIR2DL4) from R&D Systems; FES172 (KIR2DS4) and EB6 (KIR2DL1/S1) from Beckman Coulter; CHL (KIR2DL2/3/S2) from BD Pharmingen; DX9 (KIR3DL1) from BioLegend; NKVFS1 (KIR2DL1/2/3/S1/2/4) from Abcam; 5.133 (KIR3DL2) from Marco Colonna (34); and FLAG Abs from Sigma-Aldrich. Intracellular staining was performed according to the manufacturers’ instructions with Abs against Ki647 (BD Pharmingen), CCL3 (R&D Systems), and GM-CSF (BD Biosciences).

### Functional assays

Purified NK cells (CD56 positive selection using magnetic beads; Miltenyi Biotec) or mixed decidual mononuclear cells were stimulated with plate-bound anti-KIR2DS4 (179315) Abs or an isotype control for 12–48 h. After this time supernatants were removed (spun at 500 × *g* for 5 min to remove cellular contaminants) or stimulated cells were mechanically dislodged. Supernatants were analyzed using a chip-based fluorescence-linked immunosorbent assay (human cytokine Ab array G series 1000; RayBiotech) or a standard ELISA for CCL1 and XCL1 (DuoSets; R&D Systems). Cells activated cells in the presence of monensin and brefeldin A for 5 h were analyzed for surface expression of CD107a (H4A3; BD Pharmingen) or the intracellular cytokines listed above.

### Immunohistochemistry

Paraffin sections of decidual implantation sites were heat treated in 0.1 M citrate buffer for 20 min at 99.5°C. Slides were left in hot buffer for a further 20 min for Ag unmasking. Anti-XCR1 (191704 from R&D Systems) was stained in TBS with 0.1% Tween 20 for 45 min. The staining was detected with goat anti-rabbit IgG-biotin and avidin-biotin-HRP complexes (Vector Laboratories).

### Genetic typing

The case-control cohort analyzed in this study has previously been described (13). *KIR* and *HLA-C1/2* genetic typing of new patient samples was performed as in this previous study. Two-digit *HLA-C* typing was performed by the Tissue Typing facility at Addenbrookes Hospital, Cambridge, U.K.

### Statistical analysis

Statistical tests were carried out using the computational site http://vassarstats.net/, the statistical packages within GraphPad Prism v6 (GraphPad Software, La Jolla, CA), the Real Statistics Resource Pack for Excel 2010 (http://www.real-statistics.com/), and PLINK (version 1.07; http://pngu.mgh.harvard.edu/purcell/plink/) (35). The product rule was calculated by multiplying the observed frequency (Obs) of individual receptors (Obs [A] x Obs [B]) to generate the expected frequency of double-positive receptors (expected frequency [AB]). The following genetics tests were performed: a χ^2^ test and Fisher exact test with two-tailed mid-*p* adjustment, a Breslow–Day test, and a Cochran–Mantel–Haenszel test.

## Results

### *KIR2DS4wt* in epistasis with *KIR2DS1* is associated with a lower risk of pre-eclampsia

*KIR2DS4wt*, the full-length form of activating KIR2DS4, is potentially important in pregnancy as it can bind to some HLA-C allotypes (28, 36). Indeed, we previously found in a case-control cohort of European women that *KIR2DS4del* associates with increased risk of pregnancy disorders (13). The presence of *KIR2DS4wt* was neutral in this analysis. However, we only considered presence/absence of this gene and did not consider the effect of both *KIR* telomeric regions that make up the women’s genotypes. There are three possible regions: *Tel-A* containing *KIR2DS4wt*; *Tel-A* containing *KIR2DS4del*; and *Tel-B* containing *KIR2DS1* (Fig. 1A) that provides a strong protective effect (13). In this study, therefore, we reanalyzed this dataset for the effect of *KIR2DS4wt*, now controlling for the clear protective effect of *KIR2DS1*. Indeed, the presence/absence of *KIR2DS1* does alter the effect of *KIR2DS4wt*, indicative of epistasis (Breslow–Day test, *p* = 0.003). *KIR2DS4wt* is protective compared with *KIR2DS4del* in *KIR2DS1^+^* women (*p* = 5.7 × 10^−4^, odds ratio [OR] = 0.59) (Fig. 1B). This effect is not found in the absence of *KIR2DS1* (*p* = 0.83, OR = 1.0). This indicates that women who carry both *KIR2DS4wt* and *KIR2DS1* are further protected against disorders of pregnancy affecting placentation (*p* = 6.8 × 10^−5^, OR = 0.45) (Fig. 1C). Because of the similar functions and overlapping ligands of KIR2DS1 and KIR2DS4, it is likely that the epistasis detected at the statistical level reflects a biological interaction.

### KIR2DS4 is expressed by a large proportion of both pbNK and dNK cells

**FIGURE 2.**
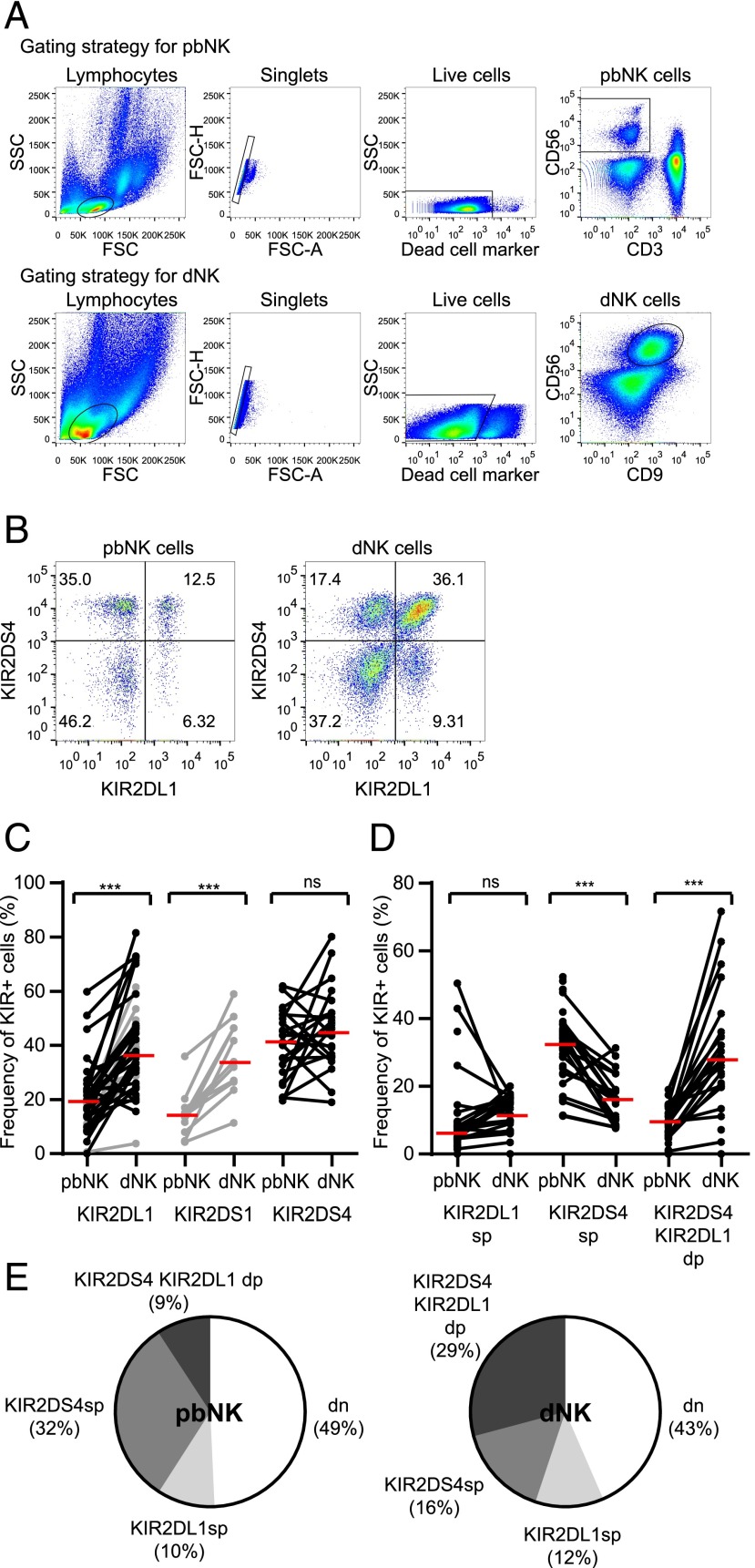
KIR2DS4 is expressed by a large proportion of both pbNK and dNK cells. (**A**) Flow cytometry plots from a typical donor showing the gating strategy for pbNK and dNK cells. (**B**) Flow cytometry plots showing KIR2DS4 and KIR2DL1 staining on pbNK and dNK cells from a representative donor. The percentage of cells in each quadrant is shown. (**C**) The proportion of KIR2DS4^+^ cells was compared for pbNK and dNK cells in matched donors (*n* = 22). The proportion of KIR2DL1^+^ (*n* = 41) and KIR2DS1^+^ (*n* = 11) NK cells is shown for comparison only. Data points for KIR2DS1 and some KIR2DL1 (shown in gray) are already published (12) and are reproduced with permission from the *Journal of Clinical Investigation*. (**D** and **E**) The proportion of NK cells from each KIR^+^ subset (single-positive [sp], double-positive [dp], or double-negative [dn] for the receptors) was compared for pbNK and dNK cells. (D) Values for individual donors. Black lines represent donors, red lines represent the median, ***p < 0.001 by Wilcoxon signed rank test. (E) The mean values for each subset are displayed as a pie chart.

Two mAbs (FES172 and 179315) were tested to confirm specificity against KIR2DS4 on cell lines expressing single KIR (Supplemental Fig. 1). The frequency of KIR2DS4^+^CD56^+^ cells is high in both dNK and pbNK cell populations (Fig. 2A–C). In contrast, both KIR2DS1 and KIR2DL1 have an increased frequency of expression in dNK cells compared with pbNK cells (12, 37, 38) and so, in accordance with the product rule, there is a higher frequency of dNK cells coexpressing these KIRs than for pbNK cells (12). This means that the proportion of cells coexpressing KIR2DS4 and other KIRs is probably different for dNK and pbNK cells. We chose to look at the distribution of KIR2DS4 relative to KIR2DL1, because KIR2DL1 is carried by almost all donors, allowing us to analyze KIR coexpression with sufficient statistical power. KIR2DL1 is also critical to our model of pregnancy disorders, as it is strongly inhibitory for HLA-C allotypes bearing C2 epitopes. Our findings (Fig. 2D, 2E) show that in pbNK cells, most KIR2DS4^+^ cells lack KIR2DL1 (Fig. 2E, mid gray segment), but in dNK cells, most KIR2DS4^+^ cells coexpress KIR2DL1 (Fig. 2E, dark gray segment). This increased coexpression obeys the product rule (Supplemental Fig. 2A), suggesting it reflects the combined frequency of KIR2DL1 and KIR2DS4. In line with this, Ki-67 staining shows that KIR^+^ dNK cells proliferate more than KIR^−^ cells, but there is no preferential proliferation by KIR2DS4^+^KIR2DL1^+^ cells compared with single-positive cells (Supplemental Fig. 2B). Therefore, in donors carrying *KIR2DS4wt*, a large proportion of dNK cells coexpresses KIR2DS4 with other KIRs that have the potential to modulate its function.

Using KIR Fc-fusion proteins, KIR2DS4 binds and responds to certain HLA-C alleles carrying both C1 and C2 epitopes (28, 36). Binding of KIR2DS4 on dNK cells to trophoblast HLA-C ligands might affect the frequency of KIR2DS4^+^ cells, but we find no difference in the proportion of dNK cells expressing KIR2DS4 when the mother or fetus carries its ligands (Supplemental Fig. 3A, 3B). There is a suggestion that allogeneic ligands affect KIR2DS4 expression, as there are fewer dNK cells expressing KIR2DS4 when the fetus alone carries a ligand, compared with the mother alone (Supplemental Fig. 3C). Given that *KIR2DS4wt* is protective in genetic case-control studies only in the presence of *KIR2DS1*, and that both are mutually exclusive on a *KIR* haplotype, protected individuals must have one copy of each gene. Therefore, we analyzed the effect of *KIR2DS4wt* copy number on frequency of expression: as two copies, as one copy in the presence of *KIR2DS4del*, or as one copy in the presence of *KIR2DS1*. KIR2DS4 frequency on dNK cells is similar in these different genetic backgrounds, suggesting that an altered frequency of KIR2DS4^+^ dNK cells in *KIR2DS1^+^KIR2DS4^+^* individuals is not the mechanism by which *KIR2DS4* provides protection against pregnancy disorders (Supplemental Fig. 3D).

### KIR2DS4 activation on dNK cells induces cytokine responses

**FIGURE 3.**
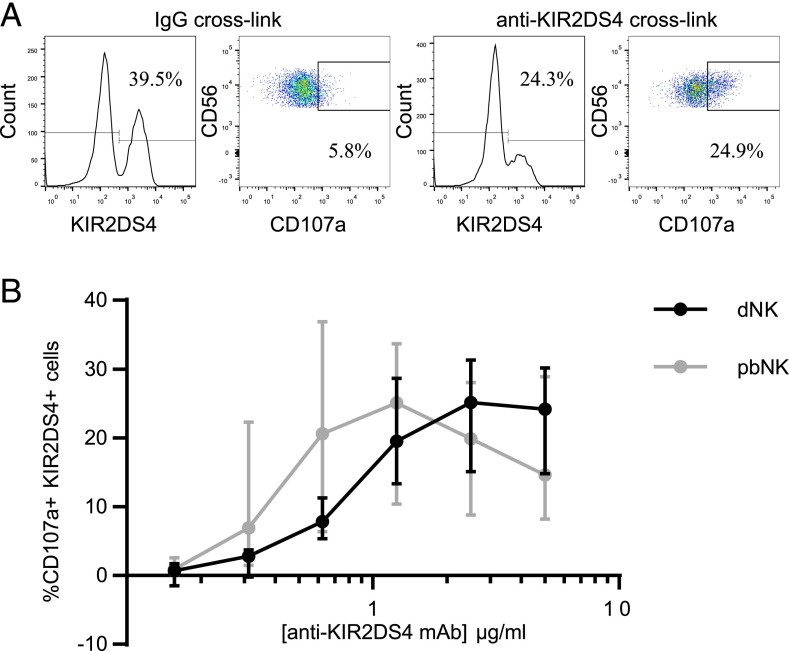
KIR2DS4 is functional on dNK cells. pbNK and dNK cells from *KIR2DS4^+^* donors were incubated in wells coated with anti-KIR2DS4 or an isotype control for 5 h in the presence of monensin. (**A**) dNK cells from a represenative donor, gated as in Fig. 2, are shown stained for KIR2DS4 and CD107a following activation with plate-bound Ab (anti-KIR2DS4 or an isotype control). (**B**) The percentage of KIR2DS4^+^ NK cells positive for CD107a upon activation was calculated by subtracting the percentage CD107a^+^ when cells were cross-linked with IgG. The extent of degranulation for a range of Ab concentrations is shown. pbNK and dNK cells were not from the same donor. Scheirer–Ray–Hare modification of Kruskal–Wallis test, effect of concentration *p* = 4.1 × 10^−10^; effect of cell type *p* = 0.24; effect of interaction *p* = 0.87. Bars represent medians and interquartile ranges.

**FIGURE 4.**
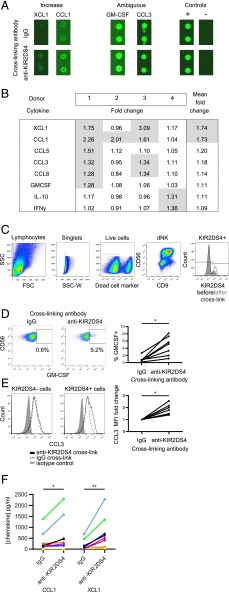
Cytokine secretion by dNK cells in response to KIR2DS4 activation. (**A** and **B**) A semiquantitative fluorescent chip-based sandwich ELISA was used to screen for 120 cytokines in supernatants taken from mixed decidual leukocytes of KIR2DS4^+^ donors (see Supplemental Table I). Leukocytes were cultured on Ab-coated plastic for 12–24 h, where the only cells to express KIR2DS4 were the dNK cells. Fluorescent spots for cytokines of interest are highlighted in (A). The cropped regions of interest are taken from different chips and different donors. They are grouped according to whether they show a >1.5-fold increase in secretion on average across all donors (Increase); secretion that was already high within the isotype control stimulation, so the screen was insensitive (Ambiguous); and control spots (Control). (B) The cytokines that were upregulated >1.25-fold upon KIR2DS4 activation in at least one of four donors tested are listed in the table. The mean fold change across all four donors is shown to the right. Values >1.25-fold are highlighted in gray. (**C**–**E**) Mixed decidual leukocytes were cultured on plastic coated with either anti-KIR2DS4 Ab or an isotype control (IgG2a) in the presence of monensin and brefeldin A. After 5 h, cells were fixed and live CD56^+^CD9^+^KIR2DS4^+^ dNK cells were identified by flow cytometry (C). Although KIR2DS4 expression reduced upon cross-linking (C), most retained KIR2DS4 expression. These KIR2DS4^+^ dNK cells were assessed for intracellular cytokines: (D) GM-CSF (*n* = 7) and (E) CCL3 (*n* = 7). (**F**) When Abs for flow cytometry were not available, purified dNK cells were cultured on Ab-coated plastic for 12–48 h and the production of CCL1 and XCL1 (*n* = 8) was detected in supernatants by commercial sandwich ELISA. Results are color coded according to donor. *p < 0.05, **p < 0.01 by Wilcoxon signed rank test.

HLA-C ligands for KIR2DS4 are shared with other NK receptors on dNK cells. To investigate the functional consequences specific to activation of KIR2DS4 alone, we used cross-linking with a specific mAb. Decidual NK cells are poor killers, as measured by chromium-release assays (6, 9, 39), but CD107a degranulation does occur in the presence of low-dose IL-15 (40) and offers a reproducible assay to quantify dNK cell activation. We find that degranulation of both pbNK and dNK cells occurs in response to increasing concentrations of anti-KIR2DS4 (Scheirer–Ray–Hare modification of Kruskal–Wallis test, effect of mAb concentration *p* = 4.1 × 10^−10^) (Fig. 3). Because cytokine responses are more physiologically relevant to human pregnancy than is degranulation (12, 41), we next analyzed the cytokines produced following KIR2DS4 stimulation of dNK cells using a semiquantitative screen of 120 cytokines (Supplemental Table I). Mixed decidual mononuclear cells were cocultured in wells coated with anti-KIR2DS4 or control IgG Ab so that contact with stromal cells is maintained, as this improves viability. We identified eight candidates that were upregulated >1.25-fold in at least one out of four donors tested (Fig. 4A, 4B). After cross-linking with anti-KIR2DS4, flow cytometry (GM-CSF and CCL3) or ELISA (XCL1 and CCL1) assays were used to validate four of these eight cytokines (Fig. 4C–F). The percentage of dNK cells positive for intracellular GM-CSF and the median fluorescence intensity for CCL3 increases (*p* < 0.05) (Fig. 4C–E) and secretion assayed by ELISA for both XCL1 (*p* < 0.01) and CCL1 (*p* < 0.05) is augmented (Fig. 4F). In summary, stimulation of KIR2DS4 on dNK cells triggers the release of cytokines, many of which are related to the cytokines upregulated at the mRNA level by dNK cells upon KIR2DS1 activation (XCL2, CCL3L3, GM-CSF, IFNG) (12), although to our knowledge this is the first time XCL1 and CCL1 have been identified as secreted by dNK cells in response to activating signals.

### Trophoblast and maternal decidual cells express receptors for newly identified cytokines produced by activated dNK cells

**FIGURE 5.**
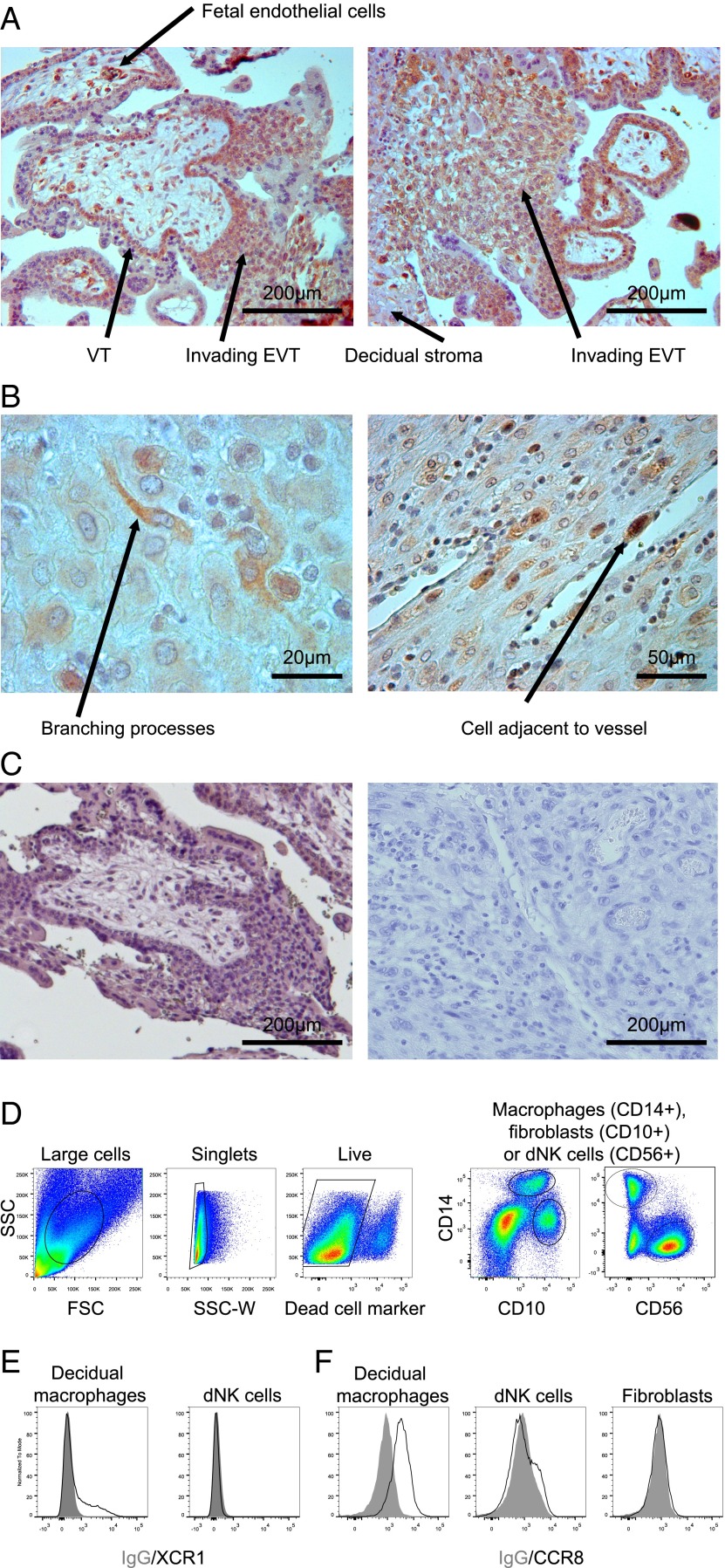
Receptors for XCL1 and CCL1 on placenta and in the pregnant uterus. (**A**-**C**) Immunohistochemical localization of XCR1, the receptor for XCL1, with DAB substrate and Carazzi's hematoxylin nuclear counterstain. (A) XCR1 was identified on trophoblast invading the pregnant uterus. (B) Within the maternal compartment, XCR1 was identified on individual cells with branching processes often adjacent to vessels. (C) Isotype control staining of trophoblast (left panel) invading the uterus (right panel). (**D**) Chemokine receptor expression on live CD14^+^ decidual macrophages was confirmed by flow cytometry for (**E**) XCR1 (*n* = 6) and (**F**) CCR8 (*n* = 5). A population of cells that did not express the receptor (dNK cells for XCR1 and fibroblasts for CCR8, because some dNK cells express low amounts of CCR8) is shown for comparison. EVT, extravillous trophoblast; VT, villous trophoblast.

Recently we have shown that GM-CSF induces migration of human primary trophoblast cells (12). CCL3 production by decidual and trophoblast cells may attract NK cells, as well as monocytes and T cells, which all bear receptors for this cytokine (42, 43). Receptors for chemokines XCL1 and CCL1 have not been described on cells at the site of placentation. We therefore stained sections of decidua and placenta and cell isolates by flow cytometry for these receptors. Several cell types within the placenta, including fetal endothelial cells, villous trophoblast, and invasive EVT express XCR1, the receptor for XCL1 (Fig. 5). Within the dNK cell–rich decidua, XCR1 is found on cells with branching processes (Fig. 5B), identified by flow cytometry as a small proportion of the CD14^+^ macrophage population (Fig. 5D, 5E). CCR8, the receptor for CCL1, is expressed by all decidual macrophages and a small proportion of dNK cells (Fig. 5F).

### KIR2DL5, the only inhibitory receptor in the *Tel-B* region, is not expressed on the surface of dNK cells

**FIGURE 6.**
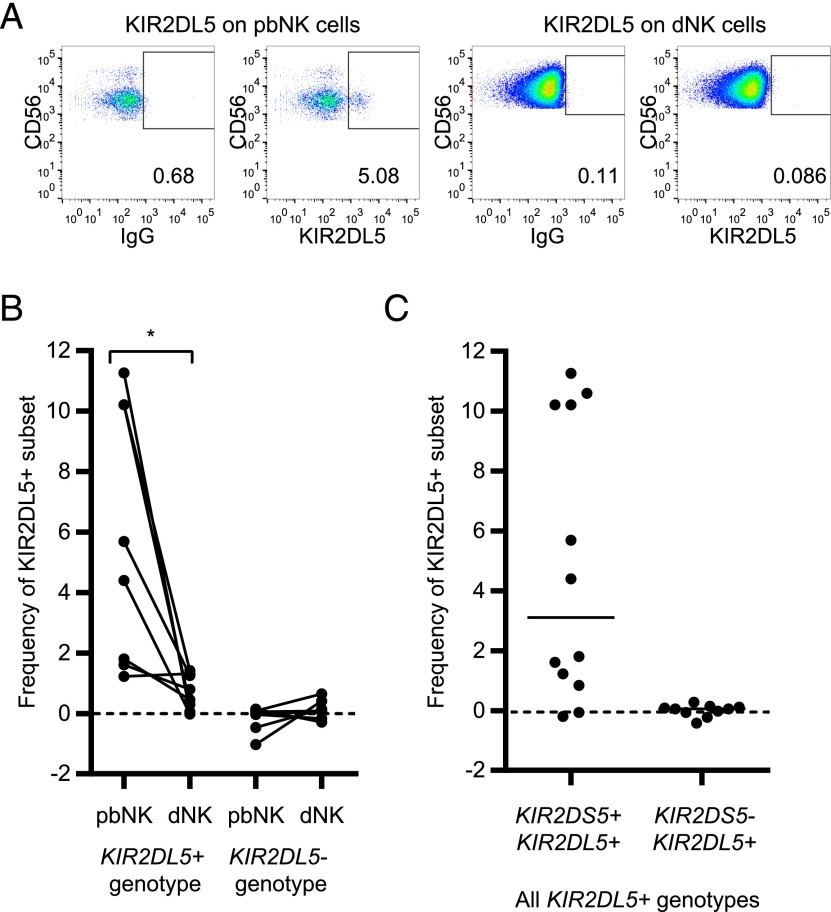
KIR2DL5 is detected by flow cytometry on the surface of pbNK cells, but not dNK cells. (**A**) Flow cytometry plots from a typical donor showing matched pbNK and dNK cells, gated as in Fig. 2, stained for KIR2DL5 (mAb UPR1) or an isotype control (IgG1). (**B**) The frequency of the KIR2DL5^+^ population was defined as percentage UPR1^+^ minus percentage IgG1^+^. The frequency of this population was measured for all pbNK and dNK cells from donors where there was UPR1^+^ staining in blood (*n* = 8). For comparison, staining of KIR2DL5^−^ donors is shown alongside (*n* = 7). Each line represents one donor. *p < 0.05 by Wilcoxon signed rank test. (**C**) All donors that carried *KIR2DL5* according to sequence-specific primer–PCR were assessed for KIR2DL5 staining of pbNK, but a positive KIR2DL5^+^ subset was only seen as part of the genotype that carried *KIR2DS5* alongside *KIR2DL5*. Lines show the median. Each dot represents one donor (*n* = 22).

In the *Tel-B* region, *KIR2DS1* is in LD with *KIR2DL5A*, which codes for an orphan inhibitory receptor. To determine whether *KIR2DL5A* affects dNK cell activation and pregnancy outcome, we first looked for expression of KIR2DL5A in dNK cells. *KIR2DL5* alleles are also found in the *Cen-B* region, where they are known as *KIR2DL5B* (Fig. 1A). To distinguish between these alleles we used Ab UPR1, which binds the most common KIR2DL5A allele in Europeans, KIR2DL5A*001 (∼30% Europeans) (33), but not KIR2DL5A*005 (∼8% Europeans) or KIR2DL5B (∼20–40% Europeans) (22). UPR1 binds KIR2DL5 and not other KIRs, which we confirmed using KIR-negative cell lines transfected with single *KIR* genes (Supplemental Fig. 1). In donors where there were detectable KIR2DL5^+^ pbNK cells, there was no surface expression of KIR2DL5 on dNK cells (Fig. 6A, 6B). The donors who expressed KIR2DL5 in blood always also carry *KIR2DS5* (Fig. 6C), which is in LD with *KIR2DS1* and KIR2DL5*001 in the *Tel-B* haplotype in Europeans, suggesting we might only detect Tel-B KIR2DL5*001 and not other KIR2DL5 allotypes. The absence of KIR2DL5 surface expression on dNK cells means it is unlikely to be affecting the activity of dNK cells, and so the protective effect of the *Tel-B* region is due to activating KIR alone.

## Discussion

We have shown that *KIR2DS4wt* is associated with lower risk of pregnancy disorders in the presence of *KIR2DS1*, representing a synergistic interaction. *KIR2DS4wt* has been linked to higher viral load and increased transmission of HIV infection (44–47), as well as with clinical outcomes in arthritis (48–50), cancer (51), and allogeneic cell transplantation (52). Because *KIRs* are in tight LD and there are confounding effects of genes on alternative haplotypes, it can be difficult to determine which particular KIR has a role in disease (53). In the present study, we have ruled out the effect of alternative haplotypes by stratifying the cohort according to the presence of *Tel-B*. A clear biological rationale for a particular KIR’s involvement can also help distinguish the effect of KIRs in tight LD. *KIR2DS4wt* is in LD with *KIR3DL1* alleles, but in the context of pregnancy, the ligand for KIR3DL1, HLA-Bw4, is not expressed by trophoblast. HLA-Bw4 can be expressed by stromal cells, so it is possible it modulates uNK cell activity. Nevertheless, KIR2DS4, known to bind to certain HLA-C allotypes expressed on trophoblast, is the likely candidate for the protective effect.

To explain how a particular KIR could affect trophoblast migration, the candidate KIR needs to be expressed by NK cells in contact with EVT in the decidua. KIR2DS4 is expressed by a large proportion of dNK cells, and its frequency of expression follows the product rule of coexpression with other KIRs. Coexpression of KIRs is relevant because the balance of activating and inhibitory signals within the cell determines activation of NK cells. Similar to KIR2DL1, KIR2DS1 has increased frequency of expression in dNK cells compared with pbNK cells. KIR2DS4wt could swing the balance in favor of activation when coexpressed with KIR2DS1 or in the context of activating cytokines, but it may fail to activate dNK cells in the absence of another activating receptor. Indeed, *KIR2DS1* may also require the presence of another activating receptor to have measurable effects on population genetics, but unlike *KIR2DS4wt*, *KIR2DS1* is in LD with two other activating receptors in Europeans. There is precedence for co-operation of activating KIRs from pregnancy and allogeneic hematopoietic stem cell transplantation, where cumulative *Cen-B* and *Tel-B* haplotypes that carry multiple activating KIR contribute to increasing beneficial effects (18, 54). Indeed, our finding that certain centromeric alleles of another different activating KIR, *KIR2DS5*, is protective against pre-eclampsia in Ugandans (21) supports this model. There is still limited evidence that KIR2DS4 responds to HLA-C molecules, but our preliminary findings suggest that the size of the KIR2DS4^+^ dNK cell subset is smaller when KIR2DS4 ligands are present in the fetus, but not the mother. This observation supports the hypothesis that KIR2DS4 does bind these HLA-C ligands on trophoblast.

Why should KIR2DS4 act as a coreceptor in this way, requiring the presence of another activating receptor? The mechanism of this co-operation remains unclear, but we can exclude some factors. First, we have shown that the frequency of expression of KIR2DS4wt on dNK cells is unaffected by the presence of *Tel-B* or *Tel-A* on the women’s other haplotype. Therefore, higher frequency of expression of KIR2DS4wt in the presence of KIR2DS1 cannot be the mechanism by which epistasis is achieved. Similarly, there is only one prevalent allele of *KIR2DS1* and functional *KIR2DS4* among Europeans (KIR2DS1*002 and KIR2DS4*001), so allelic variation on particular haplotypes is unlikely to affect the association in our European case-control cohorts. One reason for the dependence of KIR2DS4wt on the presence of KIR2DS1 could be the nature of its interaction with HLA-C molecules. Although there are functional responses of KIR2DS1^+^ NK cells upon interaction with HLA-C alleles carrying C2 epitopes ex vivo (12, 55, 56), similar responses of KIR2DS4^+^ NK cells have only been demonstrated for HLA-C*0401 (36) and HLA-A*1102 (28). The interaction of KIR2DS4 with HLA-C could be of lower avidity than that of KIR2DS1; KIR2DS4 recognition of HLA-C allotypes might be peptide-dependent, as has been shown for KIR3DS1 (23); or KIR2DS4 may be interacting with open conformers of HLA molecules (57) expressed by trophoblast. All these factors could affect the way KIR2DS4 binds to HLA-C molecules on trophoblast.

Specialized functions for pbNK and dNK cells are likely to have arisen because of the conflicting demands of disease resistance and reproductive success (3). When trying to assess the impact of KIRs in health and disease, it is necessary, therefore, to study these receptors in the species and tissue of interest. Upon triggering of KIR2DS4 with specific Abs, dNK cells degranulate and secrete cytokines, such as GM-CSF, that are known to have direct effects on trophoblast migration, and other cytokines (XCL1, CCL1, and CCL3) that have the potential to directly impact trophoblast and other cells in the decidua, including decidual macrophages. Recently, KIR2DS4 has been highlighted for promoting trogocytosis (58), a process that has been implicated in dNK cell acquisition of HLA-G from trophoblast (59). There may be several mechanisms, therefore, by which triggering of dNK cells could aid placentation.

The view that immune cells must be suppressed for successful pregnancy, both locally in the uterus and systemically, originated with Medawar (60) and the birth of transplant biology. There is now mounting evidence that for uNK cells this is not correct. We show that activation of dNK cells through KIR2DS4wt provides help to trophoblast migration and the establishment of pregnancy. Perhaps *KIR2DS5* in the *Cen-B* region may protect Africans from pre-eclampsia in the same way (21). Moreover, we find here that inhibitory receptor KIR2DL5A in the protective *Tel-B* region is not expressed by dNK cells, suggesting it does not affect pregnancy outcome. Taken together, these data support a model of generic activation of dNK cells counteracting strong inhibition by KIR2DL1 and benefitting pregnancy.

## Supplementary Material

Data Supplement
