# Supplementary material for: Activating KIR2DS4 Is Expressed by Uterine NK Cells and Contributes to Successful Pregnancy
Source: J Immunol. 2016 Nov 4;197(11):4292–300. doi: 10.4049/jimmunol.1601279 (PMC5114884; doi:10.4049/jimmunol.1601279)
Supplement: Data Supplement [file JI_1601279.zip › JI_1601279_Supplemental_Material_1.pdf]

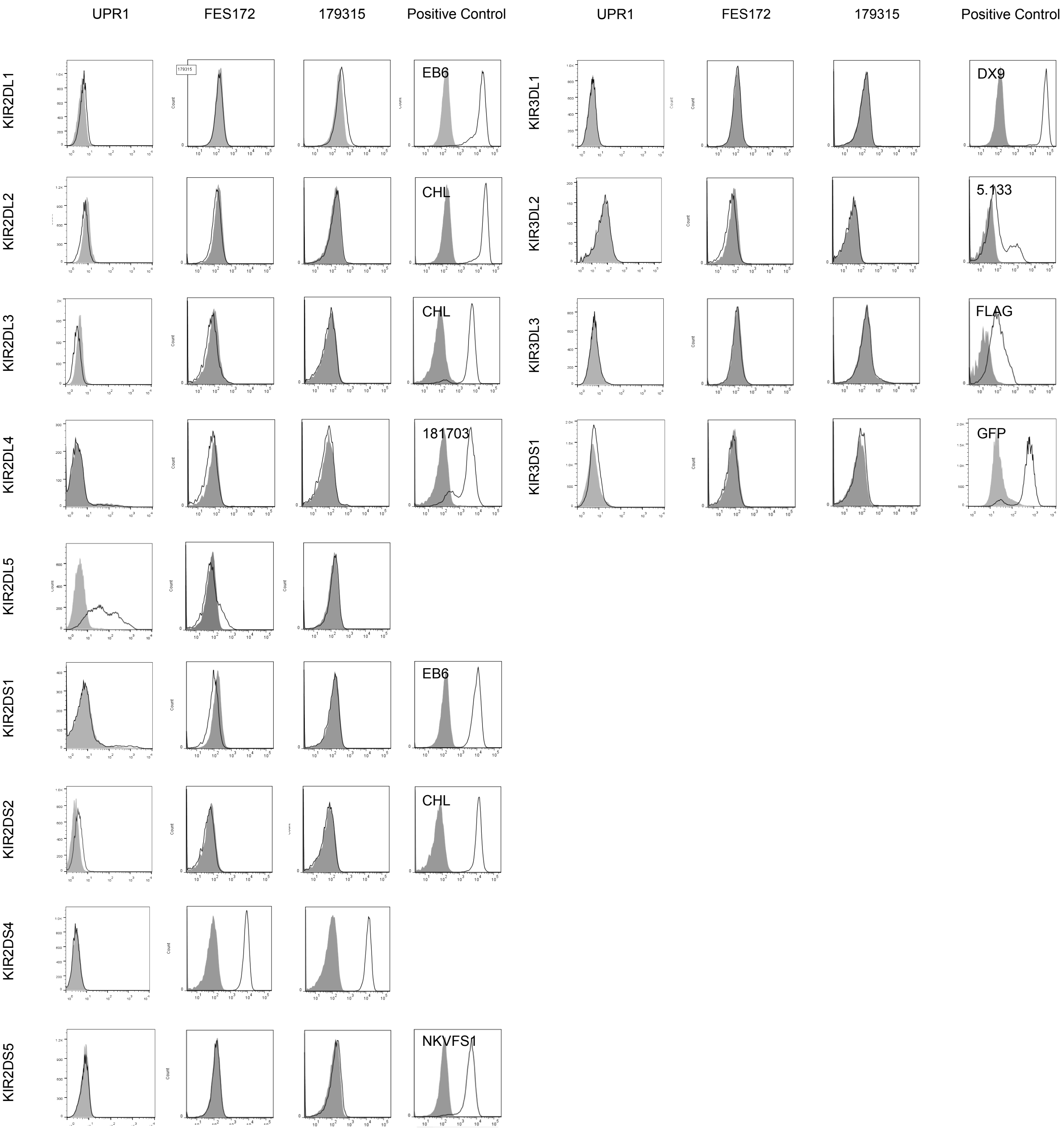

SUPPLEMENTAL FIGURE 1. Antibody UPR1 is specific for KIR2DL5 and antibodies FES172 and 179315 are specific for KIR2DS4. Cell lines expressing single KIR were stained with these three antibodies or a positive control antibody (antibody clone listed inside the plot) to check for cross-reactivity. Where transfected KIR proteins contained FLAG or GFP tags, these were used to monitor KIR expression.

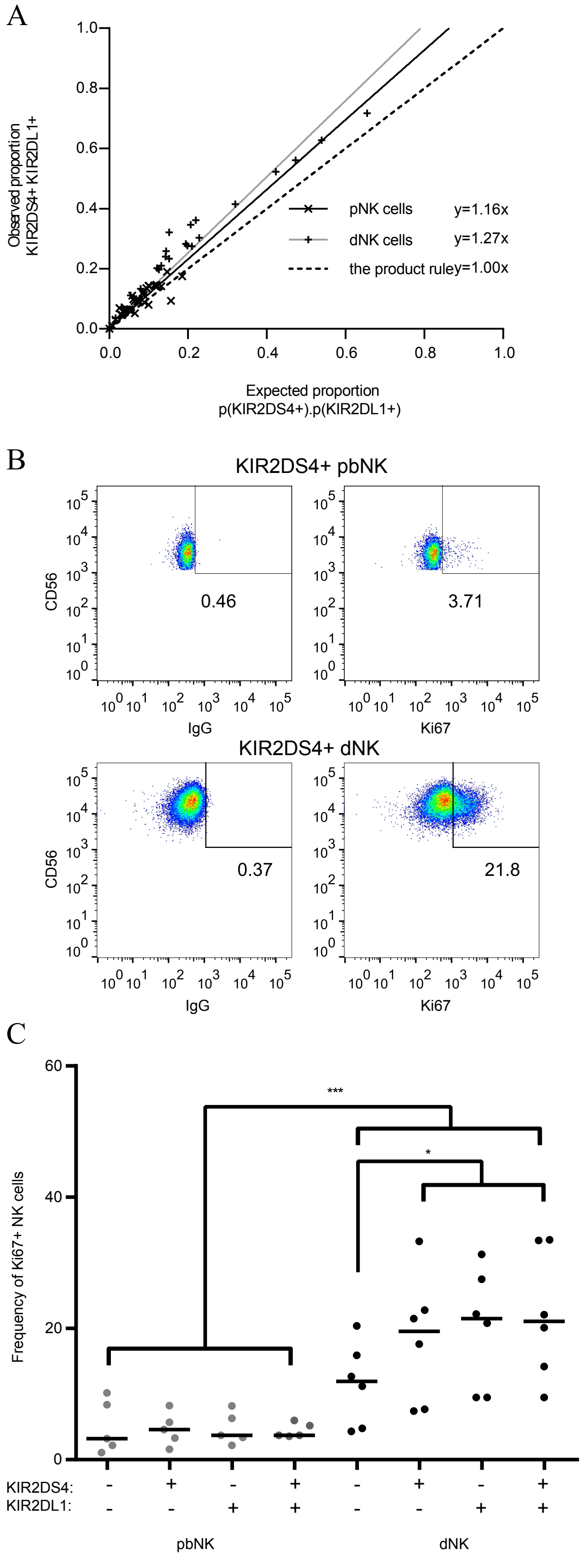

SUPPLEMENTAL FIGURE 2. The observed high frequency of KIR2DS4 co-expression with KIR2DL1 is due to the high frequency of both receptors and not a change in the product rule or proliferation. **A**. The expected proportion of KIR2DS4+ KIR2DL1+ double positive cells was calculated by multiplying the observed frequency of KIR2DS4 expression by the observed frequency of KIR2DL1 expression. This was compared with the observed frequency of KIR2DS4+ KIR2DL1+ double positive cells and a line of best fit was plotted for pbNK cells and dNK cells. Non-linear regression, the slope is the same for pbNK and dNK cells (p=0.22). **B-C**. dNK cells are proliferating if they express KIR2DS4 or KIR2DL1. **B**. Flow cytometry plots of two representative donors showing Ki67 staining or an isotype control staining of KIR2DS4+ pbNK or KIR2DS4+ dNK cells gated as in Figure 2. **C**. The frequency of Ki67+ pbNK (n=5) and dNK cells (n=6) was plotted according to the KIR subset. * $p<0.05$, *** $p<0.001$ Kruskal-Wallis test.

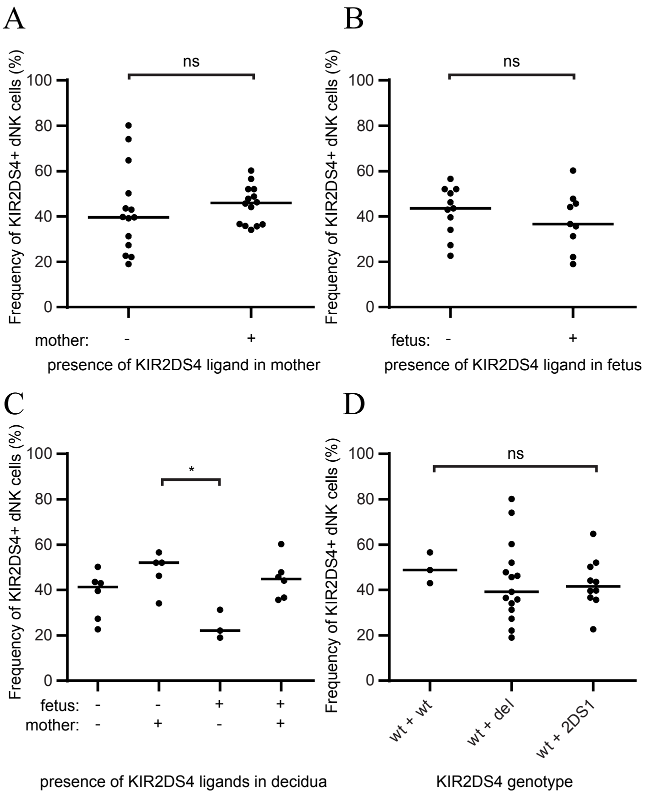

SUPPLEMENTAL FIGURE 3. The frequency of the KIR2DS4+ dNK cell subset is significantly affected by the presence of paternal HLA-C ligands in a mother that lacks a ligand, but not by the presence of other KIR genes in the mother. Maternal and fetal DNA were typed for the presence of KIR2DS4 ligands (HLA-Cw1/2/4/5/14/16) and the frequency of the KIR2DS4+ subset was plotted against whether or not there was (**A)** a ligand in the mother, (**B)** a ligand in the fetus or (**C**) a ligand in the mother, fetus or both. **(D)** shows the frequency of KIR2DS4wt expression depending on whether *KIR2DS4del* or *KIR2DS1* were also carried in the genotype. If placental tissue was not available from a particular donor to determine the genotype of the fetus, these data were included in **A** and **D**, but not **B** and **C**. (**A** and **B**) Mann Whitney test; (**C** and **D**) Kruskal-Wallis test with Dunn's multiple comparisons * $p<0.05$

Donor	1	2	3	4	
Gestational age	8	10	8	8	Mean fold
Cytokine		Fold change			change
XCL1	1.75	0.96	3.09	1.17	1.74
CCL1	2.26	2.01	1.61	1.04	1.73
PPBP	0.97	0.86	2.38	1.23	1.36
CCL13	1.13	0.92	1.69	1.67	1.35
IGFBP3	0.99	1.08	1.97	1.04	1.27
CCL5	1.51	1.12	1.10	1.05	1.20
CCL3	1.32	0.95	1.34	1.11	1.18
CCL8	1.28	0.84	1.34	1.10	1.14
CCL7	1.22	1.04	1.11	1.10	1.12
CSF2	1.28	1.08	1.06	1.03	1.11
CXCL9	1.09	1.02	1.00	1.33	1.11
IL10	1.17	0.98	0.96	1.31	1.11
IL1B	1.12	1.11	1.08	1.11	1.10
IL1A	1.08	0.87	1.02	1.41	1.10
CXCL1/CXCL2/CXCL3	1.06	0.97	1.08	1.26	1.09
CXCL1	0.78	0.71	1.02	1.86	1.09
MIF	1.16	0.93	1.03	1.23	1.09
IFNG	1.02	0.91	1.07	1.36	1.09
CCL20	1.03	1.18	1.09	1.06	1.09
IGF1R	1.04	1.12	1.12	1.08	1.09
IL13	1.17	1.08	0.92	1.18	1.09
CCL22	1.15	0.99	1.20	1.01	1.09
AREG	1.73	0.84	0.90	0.87	1.08
IL16	1.11	0.97	0.96	1.28	1.08
IGFBP4	0.94	1.05	1.20	1.12	1.08
CCL4	1.35	0.94	0.98	1.02	1.07
TNFRSF11B	0.89	0.99	0.99	1.42	1.07
CXCL5	1.09	0.89	1.04	1.25	1.07
EGFR	1.04	1.01	1.12	1.10	1.07
CSF3	1.00	0.84	1.01	1.38	1.06
TNFRSF10D	1.05	0.96	1.23	1.00	1.06
CXCL6	0.93	0.92	1.21	1.19	1.06
IL3	1.13	1.14	0.82	1.13	1.06
ANG	1.10	0.95	0.99	1.19	1.06
CCL11	1.20	0.86	1.00	1.17	1.06
CCL18	1.27	0.74	1.18	1.04	1.06
AXL	1.05	0.97	1.10	1.10	1.05
CCL26	1.17	0.97	0.85	1.23	1.05
EGF	0.98	0.99	1.10	1.14	1.05
FIGF	1.06	0.91	1.17	1.06	1.05
OSM	0.96	1.02	1.16	1.05	1.05
IL6ST	1.01	0.96	1.09	1.13	1.05
CCL25	0.99	0.94	1.17	1.07	1.04
CNTF	1.13	0.94	0.95	1.17	1.04
KITLG	1.23	0.89	0.94	1.11	1.04
IL11	1.07	0.94	0.95	1.20	1.04
BMP4	1.16	0.85	1.07	1.08	1.04
ICAM1	1.05	0.94	1.07	1.10	1.04
CCL24	1.04	0.93	1.11	1.08	1.04
IL7	1.00	0.98	1.08	1.09	1.04
PLAUR	1.05	0.94	1.04	1.11	1.04
LTA	0.90	0.76	1.04	1.43	1.03
PDGFB	1.13	0.87	1.05	1.07	1.03
CCL2	1.06	0.93	1.05	1.09	1.03
IGFBP6	1.09	0.94	1.08	1.01	1.03
IL6	1.16	1.00	0.96	1.00	1.03
TGFB3	1.01	0.91	0.95	1.24	1.03
CXCL11	1.09	0.85	1.04	1.13	1.03
FLT3LG	0.95	0.96	1.03	1.17	1.03
THPO	1.01	0.99	1.08	1.02	1.02
CCL23	1.11	0.86	1.08	1.05	1.02
PGF	1.04	0.93	1.06	1.07	1.02
IGFBP2	0.95	0.90	0.98	1.26	1.02
IL1RN	1.10	0.89	0.95	1.15	1.02
IL8	1.12	0.89	1.02	1.05	1.02
FGF7	1.14	0.93	0.85	1.16	1.02
IGF1	1.15	0.93	0.93	1.06	1.02
BDNF	0.98	0.89	1.03	1.18	1.02
CCL28	1.01	0.94	1.15	0.96	1.01
ICAM3	1.00	0.95	1.03	1.07	1.01
TNFRSF10C	1.08	1.01	0.94	1.02	1.01
FGF6	1.04	0.98	0.94	1.09	1.01
IL4	1.04	1.05	0.97	0.98	1.01
FGF2	0.93	0.92	1.08	1.12	1.01
IL5	0.95	0.94	1.03	1.11	1.01
TGFB1	1.12	0.92	0.96	1.04	1.01
HGF	0.81	0.99	1.19	1.05	1.01
GDNF	0.93	0.98	1.03	1.10	1.01
CCL15	1.09	1.06	1.08	0.80	1.01
VEGFA	1.06	0.94	0.97	1.05	1.00
IL6R	1.11	0.88	0.99	1.04	1.00

Continued…

Donor	1	2	3	4	
Gestational age	8	10	8	8	Mean fold
Cytokine		Fold change			change
IL1RL1	1.00	0.91	1.01	1.09	1.00
TNFSF18	1.03	0.95	1.02	1.02	1.00
CXCL13	0.85	1.15	1.02	0.98	1.00
NTF4	1.09	0.92	0.97	1.02	1.00
FGF9	0.96	0.97	1.04	1.03	1.00
TNFRSF1B	1.04	0.94	1.04	0.98	1.00
TIMP1	1.04	0.94	1.05	0.97	1.00
IL1R1	1.08	0.91	0.95	1.06	1.00
CCL19	1.16	0.88	0.91	1.04	1.00
IGFBP1	1.00	0.86	1.05	1.08	1.00
BMP6	0.97	0.99	0.94	1.09	1.00
TNFRSF1A	1.04	0.98	0.97	1.00	1.00
CCL27	1.02	0.94	0.98	1.05	1.00
CX3CL1	1.10	0.92	0.98	0.98	1.00
TYRO3	1.01	0.98	0.90	1.08	0.99
IL15	0.93	0.95	0.90	1.19	0.99
CXCL12	1.12	0.90	0.93	1.03	0.99
BTC	1.03	0.93	1.00	1.00	0.99
CCL17	0.96	0.89	0.84	1.26	0.99
IL2	0.99	0.92	1.00	1.03	0.99
IL17A	1.12	0.87	0.91	1.04	0.99
IL12B	1.16	1.02	0.72	0.99	0.97
ADIPOQ	1.06	0.85	1.10	0.88	0.97
FGF4	0.99	0.98	0.85	1.07	0.97
TIMP2	0.94	0.99	1.03	0.93	0.97
NTF3	1.21	0.68	1.01	0.97	0.97
NGF	0.98	0.90	0.99	1.00	0.97
AGRP	1.03	0.97	0.82	1.04	0.96
IL12A	0.99	0.94	0.90	1.02	0.96
MST1	0.90	0.97	0.96	1.00	0.96
CCL16	1.23	0.74	0.84	1.01	0.96
FAS	1.02	0.76	1.03	1.00	0.95
LEP	0.94	0.93	0.90	1.02	0.95
TNFSF14	1.07	0.87	0.72	1.12	0.94
TNFRSF18	0.81	1.00	0.91	1.03	0.94
CSF1	1.09	0.91	0.61	1.11	0.93
IL2RA	1.06	0.88	0.65	1.09	0.92
ANGPT2	0.81	0.68	1.16	1.01	0.91
TNF	0.64	0.99	0.82	1.14	0.90

Supplemental Table I: Screening cytokines secreted by decidual cells in response to KIR2DS4 activation.

A semi-quantiative fluorescent chip-based sandwich ELISA was used to screen for 120 cytokines in supernatants taken from mixed decidual mononuclear cells of KIR2DS4+ donors. Mononuclear cells were cultured on antibody-coated plastic for 12-24 hours, where the only cells to express KIR2DS4 were dNK cells. For each of the 120 cytokines tested the fold change in fluorescence intensity between the IgG stimulated and anti-KIR2DS4 stimulated supernatants is shown. Cytokines are ranked according to the mean fold change in fluorescence intensity across all four donors. The gestational age of the donors is also shown. Values greater than 1.25 fold change are highlighted in grey.
